# Food availability drives plastic self-repair response in a basal metazoan- case study on the ctenophore *Mnemiopsis leidyi* A. Agassiz 1865

**DOI:** 10.1038/s41598-017-16346-w

**Published:** 2017-11-27

**Authors:** Katharina Tissy Bading, Sarah Kaehlert, Xupeng Chi, Cornelia Jaspers, Mark Q. Martindale, Jamileh Javidpour

**Affiliations:** 1 Norwegian University of Science and Technology, Department of Biology, Bynesveien 46, 7018 Trondheim, Norway; 2 GEOMAR - Helmholtz Centre for Ocean Research Kiel, Research Division Marine Ecology, Duesternbrooker Weg 20, 24105 Kiel, Germany; 3 DTU Aqua, National Institute of Aquatic Resources, Technical University of Denmark, Kavalergaarden 6, 2920 Charlottenlund, Denmark; 4 Whitney Laboratory for Marine Bioscience, University of Florida, 9505 Ocean Shore Blvd, St. Augustine, FL 32080 USA

## Abstract

Many marine invertebrates including ctenophores are capable of extensive body regeneration when injured. However, as for the invasive ctenophore *Mnemiopsis leidyi*, there is a constant subportion of individuals not undergoing whole body regeneration but forming functionally stable half-animals instead. Yet, the driving factors of this phenomenon have not been addressed so far. This study sheds new light on how differences in food availability affect self-repair choice and regeneration success in cydippid larvae of *M*. *leidyi*. As expected, high food availability favored whole-body regeneration. However, under low food conditions half-animals became the preferential self-repair mode. Remarkably, both regenerating and half-animals showed very similar survival chances under respective food quantities. As a consequence of impaired food uptake after injury, degeneration of the digestive system would often occur indicating limited energy storage capacities. Taken together, this indicates that half-animals may represent an alternative energy-saving trajectory which implies self-repair plasticity as an adaptive trade-off between high regeneration costs and low energy storage capacities. We conclude that self-repair plasticity could lead to higher population fitness of ctenophores under adverse conditions such as in ships’ ballast water tanks which is postulated to be the major vector source for the species’ spreading around the globe.

## Introduction

Regeneration, the ability to replace missing body parts, is broadly but unevenly distributed across metazoans and is a likely result of multiple gains and losses throughout evolutionary history^1–4^. Many marine invertebrates show high regenerative capacities in response to injuries including whole-body regeneration^4^. As demonstrated for benthic invertebrate fauna, the natural frequency of injuries can be very high and is often caused through sublethal predation and physical disturbances^5^. Self-repair response can either be mere wound healing or it can be followed by regeneration of the missing body structures. The regeneration process often competes for energy resources with other life history processes such as somatic growth and reproduction leading to potential trade-offs^6,7^. Several intrinsic and external factors can influence individual regeneration success such as size, age, injury degree and food availability^6,8^. Ctenophores represent a basal metazoan phylum^9^ of generally very fragile body structure. Common sources of injuries are sublethal predation by fish and other gelatinous zooplankton^10^ as well as turbulent environments^11^. Most ctenophores show biradial symmetry and consist of an endodermal and ectodermal body layer separated by a jelly-like layer, called mesoglea^12^. The most common characteristic structures are a set of eight comb rows for means of locomotion along the oral-aboral axis and a gravity-sensing apical organ at the aboral pole. Further, they possess a digestive system with a mouth opening at the oral pole. Tentaculate ctenophores also bear a pair of tentacles for prey capture.

Under experimental conditions, ctenophores generally show remarkable healing and regenerative capacities^13^. One of the best studied species is the lobate ctenophore *Mnemiopsis leidyi* A. Agassiz 1865 which is an emerging model in evolutionary-developmental biology^14^ and has also become relevant in ecological studies due its invasion success in several marine ecosystems worldwide. The most likely vector for invasion is the transport in ballast water of ships^15^. Both the tentaculate larval and lobate adult life stage of *M. leidyi* readily regenerate and are capable of whole-body regeneration from only a body quadrant or half^13,16^. Even though whole body regeneration is the dominant self-repair trajectory of injured halves and quarters, a certain proportion of animals would often produce viable half-animals which seem to be the result of mere healing and also by partial regeneration from quarters up to this point. A definition for functional half-animals is that they only possess half the number of original body structures, i.e. one tentacle and four comb rows as well as an apical organ. They can feed, grow and undergo the normal life cycle including reproduction^13^. Further, it was shown that half-animals sometimes regenerated back to whole animals after extended periods of time or when bisected again into quarter pieces^13^. This shows that the intrinsic capacity to undergo whole-body regeneration is principally not lost. A similar self-repair mode, like the formation of half-animals in ctenophores, was recently reported for arm-amputated ephyra of the moon jelly *Aurelia aurita* ^17^. However, the formation of incomplete animals was obligate and major body regeneration did not occur.

In reviewing the literature, regeneration abilities are generally depicted as a single consistent self-repair trajectory in regenerative species and it has been suggested that little variation in regeneration trends occur among individuals^7^. Therefore, little attention has been paid so far to study self-repair plasticity in basal metazoans. Here, we aimed to investigate the role of food availability for causing plasticity in self-repair and overall regeneration success in cydippid larvae of *M. leidyi*. We hypothesized that differences in food quantity with presumable impact on internal energy reserves will affect the preferential choice of self-repair and the overall recovery success. The course of regeneration was tracked by means of a newly developed morphological body score. Furthermore, we assessed size, feeding incidence and survivability.

## Materials and Methods

### Food-dependent regeneration experiment

The experiment was carried out at the GEOMAR Helmholtz Centre for Ocean Research in Kiel, Germany during November/December 2014. Adult *M. leidyi* were collected from the Kiel Fjord (western Baltic Sea, 54°25′57.25″N 10°10′16.16″E) at ~14 °C and a salinity of ~19 psu in fall 2014. In the laboratory, the animals were acclimatized to 23 °C and 33 psu in 20 L containers filled with 0.2 µm-filtered seawater for 1–2 weeks, and fed ad libitum with lab-cultured *Acartia tonsa*. For initiating experimental cohort, animals were pooled for spawning overnight. No food was added to the cohort during the first 2 days post hatch, whereafter 200 viable and robust cydippid larvae were randomly selected to enter three subsequent experimental phases as outlined in Table S1. The remaining cohort was kept alongside at ad libitum food supply in order to replace dead larvae during phase I. During experimental phase II and III, two food quantity levels were manipulated to 100 µgC L^−1^ and 10 µgC L^−1^ in order to either support optimal growth or starvation, respectively^18^. Transfer to new incubation containers were conducted every second day. *Acartia tonsa* nauplii were used as exclusive prey source during the entire experiment. Prey concentration was determined from counting at least 3 subsamples fixed with 2% acidified Lugol. Carbon content was estimated by measuring the total length of at least 5 nauplii individuals and applying length-carbon conversion^19^. Minimum of three food controls without larvae were prepared per respective food treatment. Five days of prey pre-treatment (phase II) were applied to ensure sufficient manipulation of internal energy reserves^18^ prior to cutting (phase III). In experimental phase III, randomly selected larvae within each food treatment were bisected longitudinally through the esophageal plane while leaving the apical organ completely retained in one of the resulting halves. Uncut larvae were treated as controls. For bisection, larvae were submerged in 0.2 µm-filtered seawater inside silicone-embedded petri dishes and cut by means of very thin glass micro-capillaries which were hand pulled using a Bunsen burner. Halves with apical organ and controls were assessed every second day for a period of 10 days. Halves without the apical organ after cutting were only assessed on day 4 which is known to be sufficient time for regeneration of the apical organ^13^.

### Response assessments

Larvae were morphologically assessed under a stereomicroscope with ocular ruler and sized right before being transferred to new incubation containers. A widemouthed pipette was used for gentle handling. Pictures were taken using a Nicon CoolPix camera mounted on top of a Zeiss MZ75 stereo loupe. To track regeneration on whole-body level over time, we developed a morphological scoring system composed of an additive body score summed up from single tissue scores at distinct time points. The following four tissue types were scored: apical organ (i.e. presence of statocyst), comb row, tentacle bulb, and tentacle. Each tissue score represents the sum of four distinct tissue counts weighed by different multiplication factors. Tissue count I reflects the total number of present instances per tissue type. Count II reflects the number of those instances residing in a full-sized state, multiplied by a factor of 0.1. Count III reflects the number of those instances residing in a miniature/smaller-sized state, multiplied by a factor of 0.01. Count IV represents the number of instances in primordial state, multiplied by a factor of 0.001. The recovery of the gut was not included in the body score due to morphological variation and was therefore assessed separately (1 = flat disk shape, 2 = tube-like shape, 3 = disrupted state). Further response variables were survival (0 = dead, 1 = alive), size (total length in mm), and health rank (1 = severe morphological defect, 2 = slight morphological defect, 3 = healthy). During experimental phase I and II, size measures were performed randomly on a subgroup of approx. n = 30 larvae per respective food treatment in order to reduce experimental work load and to avoid physiological stress for the larvae during this period. Bisected larvae were sized right before cutting and not inspected again until 48 h after cutting.

### Statistical analysis

The non-linear relationship between response (body score) and explanatory variables (day and food treatment) warranted the application of a generalized additive model (GAM) considering Cox Proportional hazards distribution of errors. Size differences between treatments before cutting were analyzed by applying t-test. All statistical assumptions were met e.g. normality and constant variances and data were checked for potential outliers^20^. Statistical analyses were performed using the software R 3.0.3 (Development Core Team, 2011). For survival analysis, Kaplan–Meier estimates were calculated to compute survival curves using the survminer-package. Accidental loss of samples or samples which lived longer than 10 days post injury were treated as censored observations. Statistical significance (*P* < 0.05) was assessed by using a log-rank test. For analyzing the preferential choice of self-repair mode under different food supply, association between the categorical variables ‘half- animal’ and ‘regenerating’ was tested using Pearson’s Chi-squared test with Yates’ continuity correction (*P* < 0.05). Samples were excluded which died until day 4 post injury without showing signs of regeneration as the regenerative response fate could not be determined until day 4.

## Results

### Food pre-treatment and injury treatment

For manipulation of the nutritional state, same-aged cydippid larvae of *M. leidyi* were pre-conditioned at either high (100 μgC L^−1^) or low (10 μgC L^−1^) food concentration for a period of 5 days prior to bisection. The pre-treatment successfully yielded a significant difference in total length (*t*-test = 10.77, *P* < 0.01) between individuals at high (1.25 ± 0.24 mm, n = 59) and low (0.90 ± 0.16 mm, n = 61) food supply, respectively. Subsequently, animals were bisected through the esophageal plane using a thin hand-pulled glass needle for precise cutting. This resulted in two almost identical animal halves of which one type had the apical organ retained (+AO) and the other type was left without (−AO). Other than that, both half types were the same in possessing only four comb rows and one tentacle right after bisection. When first assessed on day 2 post-injury, the former wound sites of halves +AO were closed. Mortality was very low for both high (0%) and low food-treated halves +AO (4%) including their respective intact controls (0%). The average total length of both high and low food-treated halves +AO was reduced by 20.4 ± 16.2% (n = 27) and 31.2 ± 12% (n = 29), respectively.

### Development of assessment criteria

**Figure 1:**
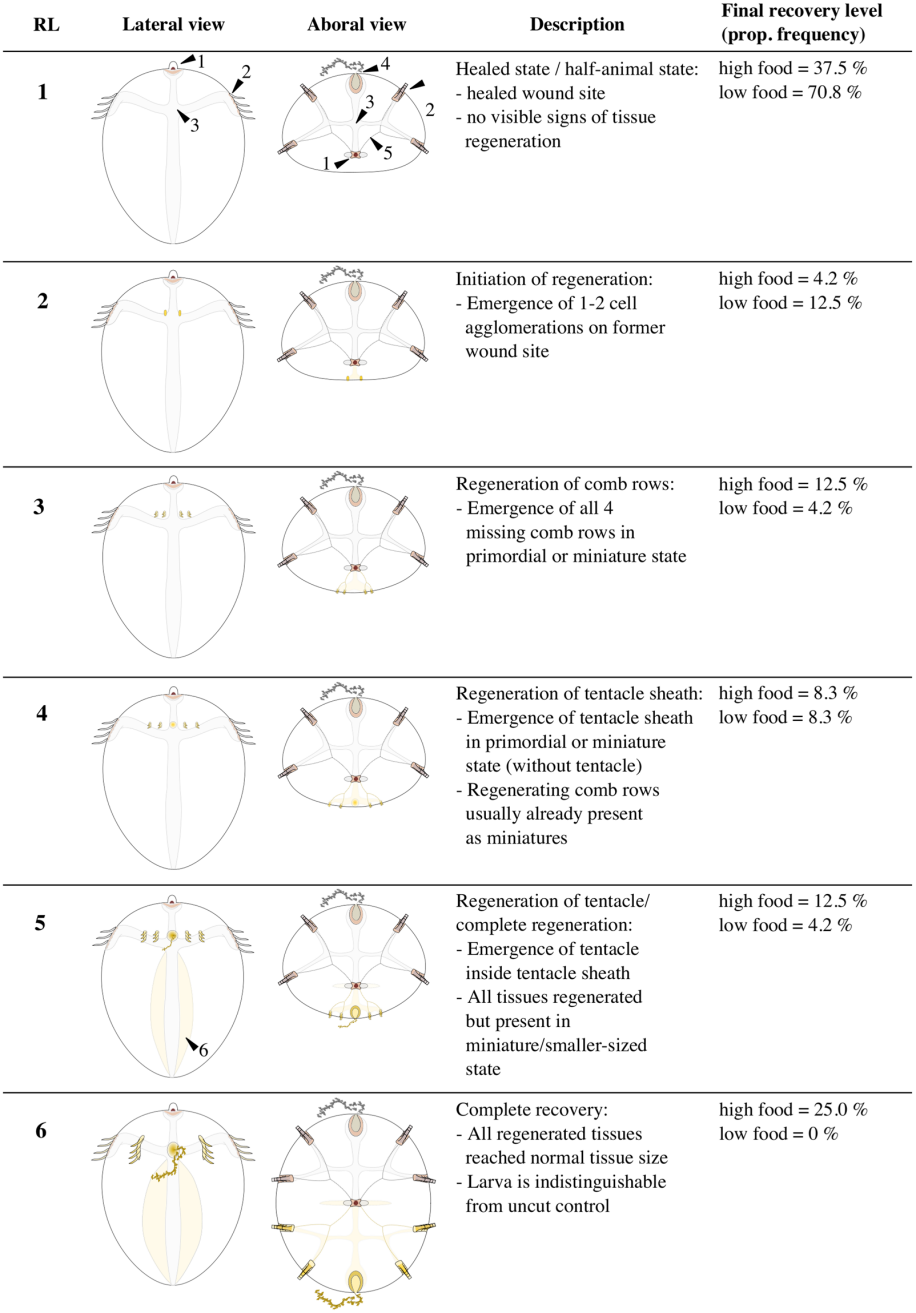
Schematic overview of whole-body regeneration in larval *M. leidyi* bisected through the esophageal plane with retained apical organ. Recovery levels were categorized according to distinct qualitative morphological recovery stages which are linked to distinct body score ranges/levels (see Fig. 2). For simplicity, tissues on the opposite body side were not depicted in lateral view. Abbreviations: recovery level (RL), proportional (prop) frequency, body score (BS), apical organ (1), comb row tissue (2), gastro-vascular system, i.e. gut and endodermal canals (3), tentacle apparatus, i.e. sheath and retractable tentacle (4), ciliated grooves connecting apical organ with comb row tissues (5), esophageal disc (6). Total n = 24 for both food levels.

**Figure 2:**
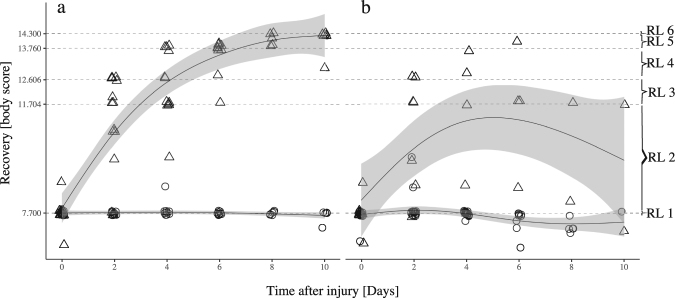
Recovery course over time under different food quantities based on a morphological body score assessment. (**a**) high and (**b**) low food quantity treatments. Initial body score after bisection (day 0) was around 7.7. Body score ranges and thresholds represent the six distinct morphological recovery levels (RL) according to Fig. 1. Generalized additive models (GAM) were used to fit the data of regenerating (triangle) and half-animals (circle) juveniles. Smoothers (method = GAM, formula = y ~ s(x, k = 5) were fitted using 95% confidence intervals.

Self-repair response of halves +AO were followed up for a period of 10 days during which the individuals were kept at the same food quantity level as during the pre-conditioning. For following the morphological recovery course of each individual, we developed an additive body score (see M&M) based on sequential tissue regeneration starting with values at 7.7 right after bisection and reaching a maximum of 14.3 when whole-body regeneration was completed. Based on the body score, intermediate recovery levels were categorized (see Fig. 1). Halves +AO that started regeneration towards whole-body regeneration were classified as “regenerating” animals whereas halves +AO that healed but showed no signs of body regeneration were classified as “half-animals” (Figure S1). Whole-body regeneration started within the first 4 days post-injury (assessment interval = 2 days). After that, half-animals were determinable. Figure 2 illustrates the significantly different courses in body score between regenerating and half-animals at both food levels respectively (GAM_(high food)_: z = −6.7, P < 0.01, GAM_(low food)_: z = −3.9, P < 0.01). While half-animals remained around a body score of 7.7 (i.e. RL 1), regenerating animals increased in body score until they regenerated completely or stagnated at an intermediate recovery level.

### Self-repair outcome

The overall proportion of regenerating and half-animals was significantly different between food treatments ($\chi$ ^2^_(1, 48)_ = 4.11, *P* = 0.04). At high food level (n = 24), the proportion of regenerating animals (62.5%) was distinctly higher than of half-animals (37.5%) whereas at low food level (n = 24) the proportion of half-animals (70.8%) was strongly increased over regenerating animals (29.2%). Whole-body regeneration of halves +AO followed the same sequential order as described in previous studies^13,21^. Figure 1 shows in simplified terms the process of whole-body regeneration from halves +AO and the respective intermediate recovery levels which individuals passed through or stopped at. After healing (RL 1), the regeneration process visibly started with the formation of 1–2 cell accumulations at the former cut site (RL 2). These primordial tissue structures subsequently split again and successively gave rise to four new comb rows (RL 3), one new tentacle sheath (RL 4) and the corresponding tentacle inside (RL5). We defined whole-body regeneration as completed when all regenerated tissues had grown out to their original tissue size (RL 6) which made the regenerated individuals undistinguishable from uncut controls. In very few cases, half-animals initiated a transient attempt to regenerate primordial comb row structures within the first 4 days which were absorbed again latest until day 6 post-injury.

### Regeneration degrees and starvation symptoms

Differences in food quantity, i.e. nutritional states, also had a significant impact on the final regeneration degrees which regenerating animals were able to reach (GAM, *t* = 7.9, *P* < 0.01). Frequencies of different intermediate recovery levels differed between high and low food treatment (Fig. 1). At high food level, the process of whole-body regeneration could be completed by 25% of the regenerating animals within 8–10 days post-injury. In contrast, regenerating animals at low food level were not able to complete whole-body regeneration. The overall body score peaked around recovery level 2 on 4 days post-injury (Fig. 2) after which it gradually decreased. Under low food level, starvation symptoms became very pronounced over time. First, it became discernible through shrinking of the digestive system. Later, also outer tissues such as comb rows, apical organ and tentacles started to shrink and gradually disintegrate which caused a decrease of body score in regenerating animals (GAM, *z* = −4.0, *P* < 0.001), but not significantly in half- animals (GAM, z = −1.8, *P* = *0.6*).

### Survivability and feeding incidence

**Figure 3:**
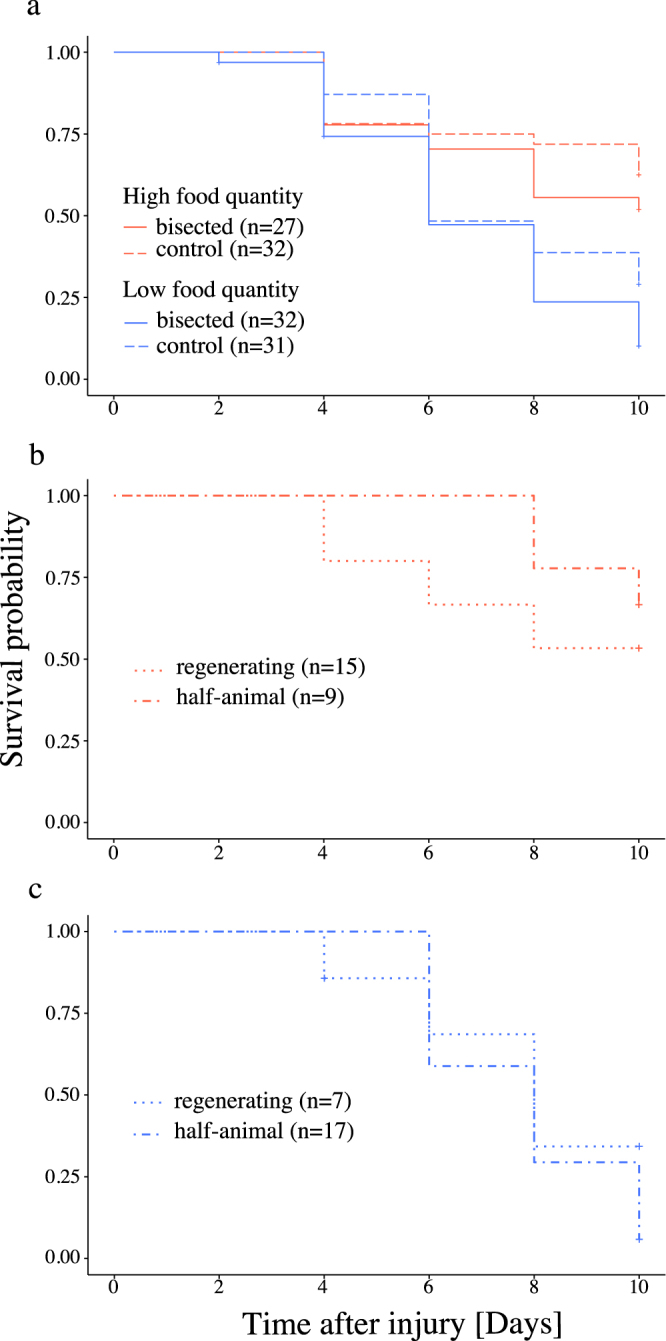
Survival curves based on Kaplan-Meier estimates of regenerating animals, half-animals and intact controls under different food quantity. (**a**) Comparison between bisected juveniles and intact controls under different food supply level. Comparison between ‘regenerating’ and ‘half-animals’ juveniles under high (**b**) and low (**c**) food supply. Cross signs indicate censored observations.

Overall survivability was significantly different between food treatments (Log-rank test, *P* < 0.01) whereas cutting treatment had no significant effect (*P* = 0.08). Interestingly, half-animals and regenerating animals seemed equally viable (Fig. 3) within the respective food treatments (*P*_*(high)*_ = 0.36; *P*_*(low)*_ = 0.41) as both showed a median survival time of 10 and 8 days post-injury at high and low food level, respectively. Despite continued food supply after injury, food ingestion at high food level was not detectable in half-animals and regenerating animals until day 6 post-injury. At low food level, bisected individuals never showed food ingestion at all (Figure S2).

### Energetic costs of apical organ regeneration

In a parallel experiment only using the halves without the apical organ (−AO), we investigated the impact of nutritional status prior to injury on the regeneration success of the apical organ. Halves –AO were kept without further food supply after bisection and assessed 4 days post-injury what is known to be the sufficient time period for regeneration of the apical organ^13^. 84.6% (n = 26) of halves which were pre-conditioned at high food level survived, of which 95.5% (n = 22) were able to regenerate the apical organ. In contrast, only 59.4% (n = 32) of halves pre-conditioned at low food level survived, of which only 63.2% (n = 19) were able to regenerate the apical organ. Concurrently, 57.9% of the low pre-conditioned survivors showed rupture or complete degeneration of the gut.

## Discussion

### Self-repair plasticity

In this study, we showed that the preferential self-repair response in bisected cydippid larvae of *M. leidyi* varies under different prey quantity regimes. Whereas regeneration attempts back to whole animals are favored under high prey quantity (63%), the formation of half-animals is the dominant self-repair trajectory under low prey quantity (71%). Previous regeneration studies on ctenophores have reported the occurrence of half-animals with variation in frequencies depending on cutting patterns^13,21,22^. However, it has not been considered as a plastic self-repair strategy in response to environmental drivers such as food availability.

As half-animals showed very similar survivability to regenerating animals, they are unlikely to be the lowest gradual outcome of initiated whole-body regeneration after early exhaustion of internal energy resources. Instead, half-animals seem to be a result of a threshold-dependent switch in self-repair upon available energy resources during wound healing. As food uptake was initially impaired after injury, energy reserves acquired prior to injury are likely to be decisive for self-repair choice. The decision-making process must take place during wound healing during which regeneration is usually initiated^23,24^. The crucial question arising is what are the underlying mechanisms which regulate the plastic switch in self-repair? So far, the molecular and cellular basis of healing and regeneration in ctenophores is mostly unknown. What is known though is that adult ctenophores maintain spatially restricted stem cell nests around tentacle base, comb rows, and the apical organ^25^. Whether or not they are involved in regeneration is not known so far, but if so, it would be interesting to investigate how mobilization of cell resources for initiating regeneration to the former cut site might be affected through nutritional differences. Another regulating mechanism could be the generation of physiological signaling molecules as a function of energy reserves that control the initiation of regeneration. However, recent literature has more and more highlighted the role of immune response and epigenetic regulation on regenerative abilities in metazoans^26^. While most half-animals of *M. leidyi* where shown to remain stable and undergo normal life cycle^13^, it was also demonstrated that they are capable to regenerate back to whole animals under certain circumstances later in life^13,27^. This means that regeneration capacity is not lost but rather temporarily silenced. As DNA methylation has been recently reported in ctenophores^28^, it is likely that epigenetic regulation plays an important role in regulating the choice of self-repair in response to available energy resources. Further studies are needed to enlighten this.

### Variation in regeneration degree under different food quantity

It has been generally known that differences in food quantity can affect the overall regeneration degree in individuals^6,29,30^ which is confirmed in our study as well. Completion of whole body regeneration in cydippid larvae of *M. leidyi* was only successful under high prey conditions and was not achieved by more than 25%. Under both food treatments, the majority of regenerating animals only reached intermediate regeneration degrees, which means that they were not able to complete whole-body regeneration due to exhaustion of internal energy reserves. Mortality among intermediate recovery degrees was observed. Reasons why intermediate recovery levels have not been pronounced in previous studies could be because of assessment differences, omission due to a different study focus or simply because a stronger food surplus was given which would have masked variation in regeneration success. However, this study shows that whole-body regeneration is likely to fail under natural conditions when food availability is not very high.

### Healing success and the role of starvation effects

Independent of injury and food quantity treatment, cydippid larvae showed 100% survival on 2 days post-injury. This highlights that *M. leidyi* larvae can easily survive severe injuries even when the nutritional state is low. Healing costs seems therefore affordable. Although low mortality is not a new finding compared to previous studies^13,21^, there had been a lack to whether nutritional differences could cause higher mortality. The initial drastic size decrease in bisected animals after injury has been observed before^21^ and is a direct effect of wound closure as the wounded halves need to round up for healing the wound. Feeding ability in bisected animals was strongly impaired after injury probably due to functional restoration of the gut. This indicates that severely injured animals can face a critical time period in which they are completely dependent on internal energy reserves in order to sustain housekeeping processes as well as healing and regeneration. Further, cydippid larvae seem to rely on the reuptake of food in order to complete regeneration up to the level where all regenerated tissue reached their original size. Although ctenophores are known to withstand food shortage for extended periods of time^18,31^, we observed that especially parts of the digestive system seem to serve as a source for energy supply as they become often quickly degraded. Gut degeneration antagonizes its functional restoration and can become critical in extreme cases such as for halves attempting to regenerate the apical organ. Here, we often observed rupture or complete disintegration of the gut when the animals were pre-treated at low food supply prior to injury. As gut disintegration was observed on 4 days post-injury, this suggests that the regeneration of the apical organ as such is probably a quite energetically demanding regeneration step, which can potentially cause early mortality when food conditions are not sufficient. Interestingly, this could also provide an explanation why previous studies had shown that halves without the apical organ retained after injury would be less likely to complete whole body regeneration^13,21^ but instead rather remain as half-animals after regeneration of the apical organ. It is probably important to regenerate the apical organ in order to regain full locomotive functionality. However, afterwards it could be energetically often more favorable to remain as half-animal as nutritional reserves might be already critically low for supporting whole-body regeneration. Overall, this shows that not only the nutritional state gained prior to injury is relevant for recovery success from severe injuries but also the initial injury degree itself. Here, the additional presence or absence of the apical organ seems to be relevant.

### Constrains of this study

Previous studies indicated a prey concentration of around 100 µgC L^−1^ to be sufficient for supporting growth^18,32^. However, *M. leidyi* larvae in this study only reached an average total length around 1.1 mm after 17 days post hatch, which indicates low growth rates for larval *M. leidyi*. Likely sources for growth limitation could have been the chosen container type and incubation volume. We used rectangular culture flasks with canted necks. Even though the samples were kept in darkness, the rectangular shape could have caused unwanted prey aggregations in corners and hence reduction in prey availability. An incubation volume of 50 ml was calculated based on literature^33^ but might have underestimated the available space for full tentacle extension in larval *M. leidyi*. However, given the fact that food availability is variable in nature, our study is likely to represent realistic self-repair outcomes under suboptimal growth conditions in nature. A further limitation of this study was the restricted number of samples which were processable. As ctenophores are very delicate organisms, it required careful handling and hence extra time in order to avoid additional stress and unwanted injuries. Due to the restricted sample number, it was not feasible to remove samples for additional physiological measures, such as volumetric measurements. A further limitation is that there seems to be no feasible approach how to measure initial differences in energy content of bisected halves for which it was not known until finally 4 days post-injury whether they would regenerate or remain as half-animals. Alternative measures for proof will need to be established.

### Ecological relevance of self-repair plasiticity

Although half-animals and possibly other forms of incomplete ctenophores are likely to occur frequently in nature^10,27^, their ecological relevance on population and community-level is not well understood. Common injury sources are sublethal predation by fish and other gelatinous zooplankton as well as probably turbulent environments^11,27^. Natural frequencies of injury and subsequent self-repair are difficult to assess in the field, as injury rates can vary strongly in time and space, and previous injuries can be masked by rapid regeneration^5^. As shown for benthic invertebrate communities, injury frequencies and individual capacity of healing and regeneration can affect community dynamics^5^. If this also applies to pelagic communities needs still to be resolved. Further laboratory experiments will be important to investigate the impact of ecological factors on the formation and retention time of half-animals under various ecological conditions, and to study potential trade-offs between whole body regeneration and half-animal formation. Another important ecological scenario in which self-repair plasticity of *M. leidyi* could play a role is the transport in ballast water tanks of ships which is postulated to be the cause for several unwanted introductions of *M. leidyi* in various ecosystems worldwide^15^. Major life history traits generally associated with its invasion success are self-fertilizing hermaphroditism, fast growth and high reproduction rates as well as tolerance to abiotic and biotic stressors^10,15^. Interestingly, the role of healing and regeneration has been largely neglected so far although the vector transport in ballast water entails pumping processes during uptake and discharge with potentially detrimental effect on animals’ body integrity. Here, self-repair plasticity may facilitate higher changes of survival and recovery of *M. leidyi* inside the ballast tanks and when discharged into new environments.

### Evolutionary context

This study supports a subtle shift in perspective on how to view the emergence and presence of high regeneration capacities in basal metazoans. As demonstrated for *M. leidyi*, the attempt of whole body regeneration is not a single axiomatic self-repair trajectory irrespective of environmental conditions. Instead, individuals are likely to adopt their self-repair trajectory according to environmental food conditions. This might be an important trade-off considering that ctenophores show a generally low carbon content^34^ with seemingly no explicit storage tissue as this could most likely not guarantee reliable regeneration success under highly variable food conditions in nature. We therefore hypothesize that self-repair plasticity is a possible adaptive trait in order to maximize population fitness in variable environments.

### Future directions

Having its genome sequenced, *M. leidyi* represents a very suitable model organism to investigate the underlying cellular and molecular mechanisms regulating self-repair plasticity in response to variable environments. Due to generally little variation in regeneration tendencies, there is a lack in suitable study models that would allow a direct comparison of trade-off between regenerating and non-regenerating animals under given environmental conditions^7^. The ctenophore *M. leidyi* seems to fulfil this criterion while holding a key position at the phylogenetic base of the metazoan tree. Therefore, future studies on self-repair plasticity in *M. leidyi* may give important insights into the evolution of regeneration, its regulation, and how energy-related trade-offs may have shaped its presence and absence of regeneration across metazoans^4,7,35^.

### Data Accessibility

Raw data underlying this paper will be available at PANGAEA.

## Electronic supplementary material

Supplementary Information
