# Supplementary material for: Food availability drives plastic self-repair response in a basal metazoan- case study on the ctenophore Mnemiopsis leidyi A. Agassiz 1865
Source: Sci Rep. 2017 Nov 27;7:16419. doi: 10.1038/s41598-017-16346-w (PMC5703731; doi:10.1038/s41598-017-16346-w)
Supplement: Supplementary file 1 — Supplementary Information [file 41598_2017_16346_MOESM1_ESM.pdf]

Supplementary Information

Title: Food availability drives plastic self-repair response in a basal metazoan- case study on the ctenophore *Mnemiopsis leidyi* A. Agassiz 1865

Authors: Katharina Tissy Bading[a]*, Sarah Kaehlert[b], Xupeng Chi[b], Cornelia Jaspers[b,c], Mark Q. Martindale[d], and Jamileh Javidpour[b]*

Authors affiliations: [a]Norwegian University of Science and Technology, Department of Biology, Bynesveien 46, 7018 Trondheim, Norway; [b]GEOMAR - Helmholtz Centre for Ocean Research Kiel, Research Division Marine Ecology, Duesternbrooker Weg 20, 24105 Kiel, Germany; [c]DTU Aqua, National Institute of Aquatic Resources, Technical University of Denmark, Kavalergaarden 6, 2920 Charlottenlund, Denmark; [d]Whitney Laboratory for Marine Bioscience, University of Florida, 9505 Ocean Shore Blvd, St. Augustine, FL 32080, USA

* **Corresponding author:** jjavid@geomar.de

Supplementary information includes:

1. Supplementary figures and table
2. Supplementary figure and table legends

Figure S1

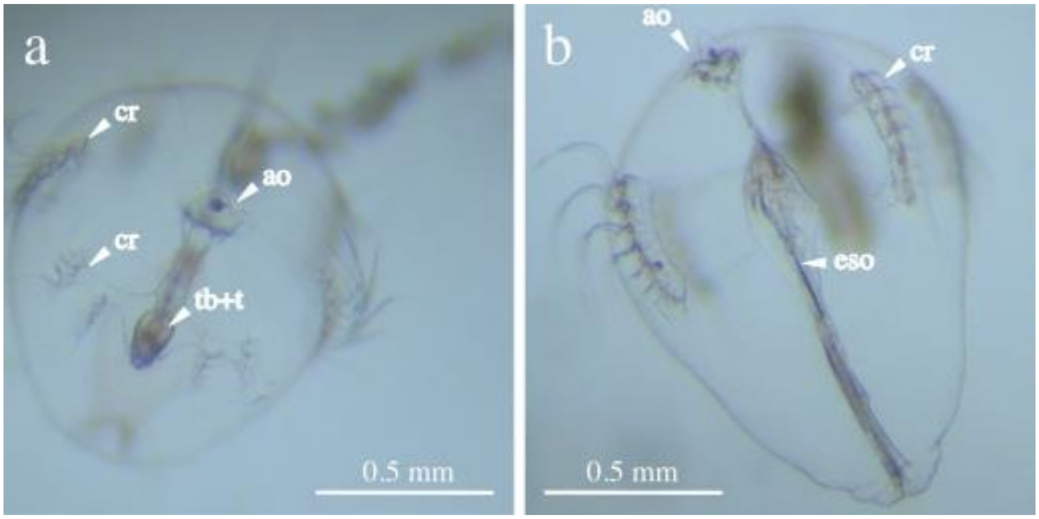

Figure S2

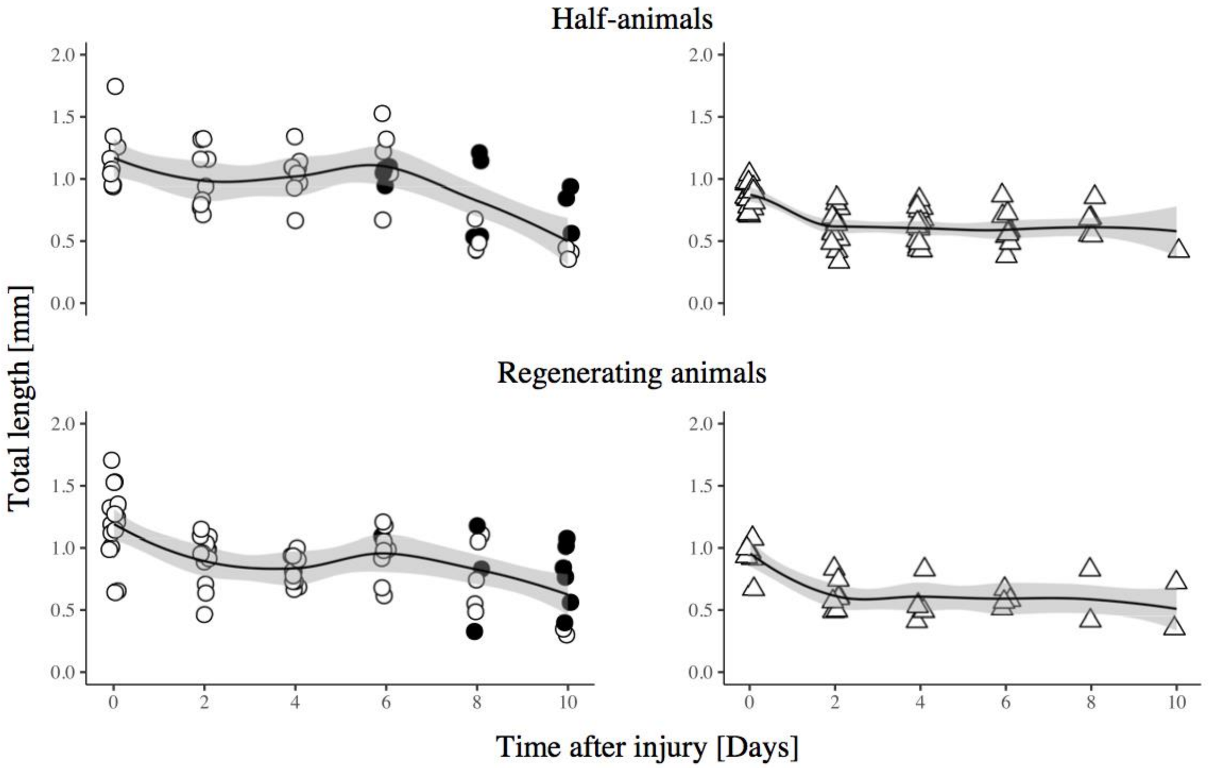
Half-animals
Regenerating animals
Total length [mm]
Time after injury [Days]
2.0
1.5
1.0
0.5
0.0
0
2
4
6
8
10

Table S1

Exp. phase	Time (days post hatch)	Food quantity ($\mu gC\ L^{-1}$)	Integrity status	Sample size (n)	Container volume (ml)	Light:dark cycle (hours:hours)
I – rearing	2 – 17	100	-	200	20 (day 1- 9)<br>50 (day 9-17)	12:12
II – pre-conditioning	17 – 22	100	-	74	50	0:24
		10	-	108		
III – recovery (post-bisection)	22 – 32	100 (continued)	½ +AO	27	50	0:24
			control	32		
		10 (continued)	½ +AO	31		
			control	32		
		0 (pre-conditioned at 100)	½ -AO	26		
			control	5		
		0 (pre-conditioned at 10)	½ -AO	32		
			control	3		

Figure S1- Examples of self-repair trajectories in bisected cydippid larvae of *M. leidyi*. (*A*) Aboral view on bisected larvae which undergoes whole body regeneration. (*B*) Lateral view on half-animal which shows no signs of tissue regeneration on former cut site. Abbreviations: apical organ (ao), comb rows (cr), tentacle bulb and tentacle (tb+t), esophagous (eso).

Figure S2- Changes in total length and ability of food ingestion over time in half-animals and regenerating animals of larval *M. leidyi*. Data points of high and low food treatment are symboled as circles and triangles, respectively. Filled data points indicate the presence of ingested food at the time point of assessment. Loess smoothers were fitted with 95% confidence bands (grey area)

Table S1- Experimental overview of food-dependent regeneration experiment. Abbreviations: apical organ (ao), intact larvae (control), bisected larvae (1/2) with/out apical organ (±AO).
